# Biostimulants for the Regulation of Reactive Oxygen Species Metabolism in Plants under Abiotic Stress

**DOI:** 10.3390/cells10102537

**Published:** 2021-09-25

**Authors:** Mirza Hasanuzzaman, Khursheda Parvin, Kirti Bardhan, Kamrun Nahar, Taufika Islam Anee, Abdul Awal Chowdhury Masud, Vasileios Fotopoulos

**Affiliations:** 1 Department of Agronomy, Faculty of Agriculture, Sher-e-Bangla Agricultural University, Sher-e-Bangla Nagar, Dhaka 1207, Bangladesh; taufika_islam@sau.edu.bd (T.I.A.); chy.masud3844@gmail.com (A.A.C.M.); 2 Department of Horticulture, Faculty of Agriculture, Sher-e-Bangla Agricultural University, Sher-e-Bangla Nagar, Dhaka 1207, Bangladesh; hirasau@gmail.com; 3 Department of Basic Sciences and Humanities, Navsari Agricultural University, Navsari 396450, India; kirtivardhan@nau.in; 4 Department of Agricultural Botany, Faculty of Agriculture, Sher-e-Bangla Agricultural University, Sher-e-Bangla Nagar, Dhaka 1207, Bangladesh; knahar84@yahoo.com; 5 Department of Agricultural Sciences, Biotechnology & Food Science, Cyprus University of Technology, P.O. Box 50329, Lemesos 3603, Cyprus

**Keywords:** antioxidant defense, organic amendments, phenolic compounds, phytohormones, trace elements, stress tolerance, sustainable agriculture

## Abstract

Global food security for a growing population with finite resources is often challenged by multiple, simultaneously occurring on-farm abiotic stresses (i.e., drought, salinity, low and high temperature, waterlogging, metal toxicity, etc.) due to climatic uncertainties and variability. Breeding for multiple stress tolerance is a long-term solution, though developing multiple-stress-tolerant crop varieties is still a challenge. Generation of reactive oxygen species in plant cells is a common response under diverse multiple abiotic stresses which play dual role of signaling molecules or damaging agents depending on concentration. Thus, a delicate balance of reactive oxygen species generation under stress may improve crop health, which depends on the natural antioxidant defense system of the plants. Biostimulants represent a promising type of environment-friendly formulation based on natural products that are frequently used exogenously to enhance abiotic stress tolerance. In this review, we illustrate the potential of diverse biostimulants on the activity of the antioxidant defense system of major crop plants under stress conditions and their other roles in the management of abiotic stresses. Biostimulants have the potential to overcome oxidative stress, though their wider applicability is tightly regulated by dose, crop growth stage, variety and type of biostimulants. However, these limitations can be overcome with the understanding of biostimulants’ interaction with ROS signaling and the antioxidant defense system of the plants.

## 1. Introduction

Access, availability and affordability of food to everyone are crucial for world peace and prosperity. Since 1900, scientific crop production and breeding have made enormous growth in agricultural production and productivity by developing high-yielding varieties and agronomic practices. Nevertheless, food is still not sufficiently available to all, and we are far from achieving the UN sustainable development goal of zero hunger by 2030, when it is expected that 330 million will be starving in Asia and 433 million people in Africa will be undernourished [1]. The world population is continuously growing and will reach 9.6 billion in 2050 and 10.9 billion in 2100 [2], while food demand will increase by 60% by 2050 which will not be met by further increasing agricultural land [3]. In addition, yield ceiling or reduction is observed in major crops [4], due to region-specific soil, water and climatic variability [5,6]. For instance, annual drought and high temperature events substantially decrease global cereal average yield [7].

Adverse environmental conditions trigger complex plant responses which are regulated by signaling molecules, transcription factors, genes and defense system which help in coping with adverse stress effects. Individual abiotic stress triggers individual as well as common responses with other stresses, which may be synergistic or antagonistic [8]. Thus, crosstalk and overlapping between signaling pathways is observed. One of the common responses under stress conditions is the generation of reactive oxygen species (ROS) such as singlet oxygen (^1^O_2_), superoxide anions (O_2_^•−^), hydrogen peroxide (H_2_O_2_) and hydroxyl radicals (OH^•^) etc. [9]. The ROS interact with phytohormones and influence multiple signaling pathways [10]. Their involvement in signaling pathways implies that ROS must be kept under non-toxic levels by a coordinated balance of production and elimination.

Plant growth and development can be adjusted by exogenous application of various chemicals and biostimulants, and these approaches are gaining considerable attention because of their natural origin and have a promising role in improving crop stress tolerance indicated by different physiological attributes like photosynthesis and nutrient assimilation [11]. Moreover, contrary to the development of multiple stress tolerant varieties, the use of biostimulants is a sustainable solution for enhancing crop health under abiotic stress and helps in managing climate-resilient farms [12]. This review discusses the function of biostimulants towards the optimization of ROS homeostasis in different crops for the management of climate change-related abiotic stress conditions. The focus is mainly on broadly examined biostimulants in the form of a mixture of microorganisms and natural products, such as protein hydrolysates, humic and fulvic acids, basic nutrients, and extracts from plants and algae. This focus is due to the fact that the number of potential compounds and formulations with biostimulatory activity is vast, while other commonly used components such as plant nutrients mainly act as indirect biostimulants by having direct nutritional effects and are mainly used as biofertilizers.

## 2. Plant Abiotic Stresses and Reactive Oxygen Species

Plant responses to abiotic stresses are results of interplay between different signaling molecules and hormonal balance which dictate crop performance by altering metabolism and gene expression levels [13]. ROS refers to short-lived, highly reactive reduced-state oxygen atoms or molecules, which, if produced in excess, oxidize proteins, lipids, DNA and RNA and lead to cell death [14]. Here, we aim to summarize ROS generation under abiotic stresses and its detrimental effects, and the antioxidant protective mechanisms of plants.

### 2.1. ROS Generation under Abiotic Stresses

**Figure 1:**
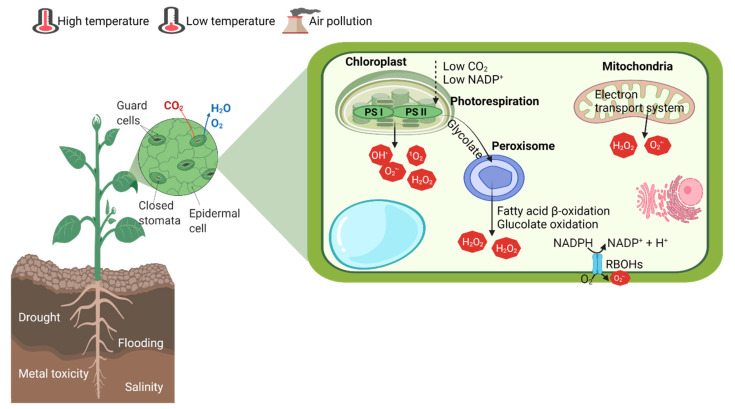
Principal cellular sites of ROS generation during abiotic stresses. Created with BioRender.com (accessed on 15 May 2021).

ROS are synthesized in most of the cellular compartments, including chloroplast, mitochondria, peroxisomes, plasma membrane, apoplast, cell wall and endoplasmic reticulum [15]. However, major sites of ROS generation are the chloroplasts, peroxisomes and the mitochondrial respiratory electron transport system. Stomatal closure is a common adaptive strategy to reduce water loss under abiotic stresses, though it also reduces CO_2_ flux and net photosynthesis. This causes direct as well as indirect effects on ROS generation during stress. Low internal CO_2_ concentration limits the rate of Calvin cycle reactions and limits the generation of NADP^+^. This would increase the electron loads in photosynthetic electron transport system. The photosystem I and photosystem II in the thylakoid membrane are major sites of ROS generation. These excess electrons reach O_2_ by the Mehler reaction [16]. For instance, in wheat under water stress conditions, 30% of electrons are diverted to the Mehler reaction [17]. Similarly, salinity also decreases stomatal conductance, which is due to apoplast alkalization, abscisic acid (ABA) accumulation and ion redistribution [18]. The reduced CO_2_ flux favors photorespiration in C_3_ plants and H_2_O_2_ is generated in peroxisomes as a result [19]. Peroxisomes are also important sites for ROS generation by photorespiration, fatty acid β-oxidation and enzymatic oxidation reactions [20]. Membrane-bound NAD(P)H oxidase, also known as respiratory burst oxidase homologues (RBOHs), is/are also known to contribute to ROS generation during stress [21] (Figure 1). Abiotic stresses such as flooding, drought, salinity, light, heavy metal toxicity, and high and low temperatures favor photorespiration and produce glycolate, which moves into peroxisomes and glycolate oxidase, leading to H_2_O_2_ generation [22]. In mitochondria, a respiratory complex of the electron transport system is the site of ROS generation [23]. Under drought, salinity, and chilling stress, mitochondrial ROS generation is observed [21,22,24,25]. However, the initial overproduction of ROS does not cause instant harm to the cellular mechanism, even if it may be beneficial to the plants. Plants sense this rise as an alarming signal which triggers adaptive responses [26,27]. For instance, under stress, ABA-induced H_2_O_2_ production in guard cells, which in turn activate membrane-bound Ca^2+^ channels, causes ABA-driven stomatal closure [28,29].

### 2.2. Generation of ROS and Its Consequence

**Figure 2:**
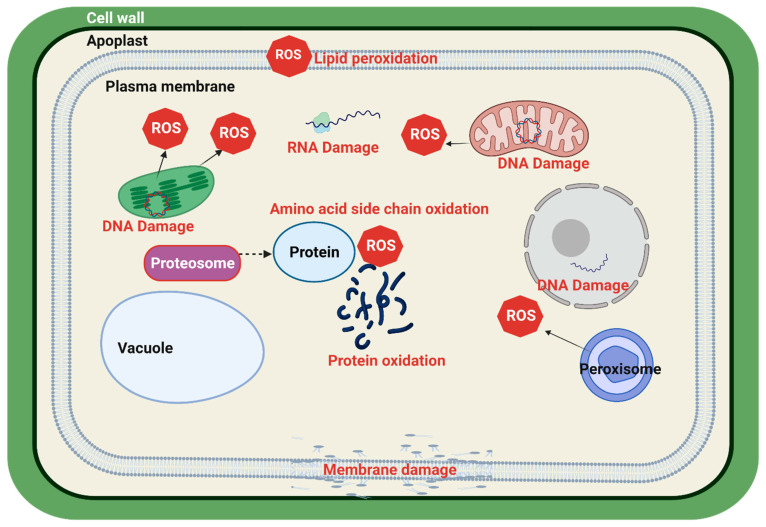
Major effects of oxidative stress on cellular machinery. Created with BioRender.com (accessed on 15 May 2021).

Overproduction of ROS causes oxidative burst in the cell and initiates protein oxidation, lipid peroxidation, changes membrane permeability, causes DNA and RNA damage, and in severe cases, leads to cell death. Protein oxidation is a direct effect of ROS where side chains of amino acids are oxidized. This damage alters the protein functionality [24]. ROS also causes indirect oxidation of proteins which is mediated by products of lipid peroxidation (Figure 2) [24,30]. These changes make proteins prone to proteolytic reactions mediated by proteasome [24]. Some damages to proteins are irreversible such as lysine and arginine carbonylation, tyrosine and tryptophan nitration, dityrosine formation, and protein–protein cross-linking, thus leading to activity loss, while others such as glutathionylation and *S*-nitrosylation are reversible [31]. Oxidation of heat shock and late embryogenesis abundant (LEA) proteins may reduce plant tolerance to stressful environments [32]. Unsaturated fatty acids of the cellular membrane are highly sensitive to ROS damage [31]. ROS break the phospholipid bi-layer by breaking ester bonds between glycerol and fatty acids [22,33]. Oxygen bursts also lead to altered gene expression and protein synthesis by damaging nucleic acids [33,34]. Chloroplasts and mitochondrial DNA are more sensitive to ROS damage than nuclear DNA, as these organelles are primary sites of ROS generation and their nucleic material is free from histones and other protein complexes. ROS alter nucleotide bases, oxidize sugar molecules and break the DNA strand [22,35]. ROS overproduction severely interrupts cellular activity and functionality of biomolecules, so it is crucial to overcome these effects either by enhancing the intrinsic antioxidant defense or repairing the damage.

### 2.3. Antioxidant Defense System in Plants

Cellular ROS homeostasis is essential for plants due to the dual role of ROS. They act as signaling molecules that regulate plant growth and development and acclimatize plants under stress while causing detrimental effects on cellular metabolism when their concentration increases under stress conditions [36,37]. Thus, plants are equipped with a complex antioxidant system to regulate the balance of cellular redox potential. However, the strength and potentiality of antioxidative protection is highly species- and genotype-dependent [38,39,40]. Plant antioxidative defense consists of enzymatic and non-enzymatic components which scavenge or inhibit the oxidative action of ROS to prevent/delay cellular damage [41]. The enzymatic antioxidant defense is comprised of superoxide dismutase (SOD), catalase (CAT), ascorbate peroxidase (APX), non-specific peroxidase (POX), monodehydroascorbate reductase (MDHAR), dehydroascorbate reductase (DHAR), glutathione *S*-transferase (GST), glutathione peroxidase (GPX), alternative oxidase (AOX), and peroxiredoxin (Prx) while ascorbic acid (AsA), glutathione (GSH)**,** carotenoids, flavonoids, and α-tocopherol comprise the non-enzymatic antioxidative defense line [9,42]. Enzymatic defense plays a major role in protection against ROS. Superoxide radicals are converted into H_2_O_2_ by the activity of SOD, while APX, GPX, and CAT are responsible for further conversion into the water and APX shows its activity via ascorbate-glutathione (AsA-GSH) cycle in cytosol, chloroplast, mitochondria, and peroxisomes [43]. AsA is a strong non-enzymatic antioxidant that is present in almost all cellular compartments, including apoplasts [22]. AsA scavenges OH^•^, O_2_^•−^, ^1^O_2_, and H_2_O_2_ into water through AsA-GSH cycle [10]. GSH non-enzymatically neutralizes OH^•^, O_2_^•−^, and ^1^O_2_ and scavenges H_2_O_2_. GSH is important in the sense that it participates in the regeneration of AsA via the AsA-GSH cycle [10]. Carotenoids, phenols, and α-tocopherol all play important roles in protecting thylakoid membrane and other cellular membranes from lipid peroxidation [9,14]. Thus, a critical balance between ROS generation and antioxidant activities is crucial for crop health under abiotic stresses.

## 3. Biostimulants: Types, Mode of Action and Methods of Applications

**Figure 3:**
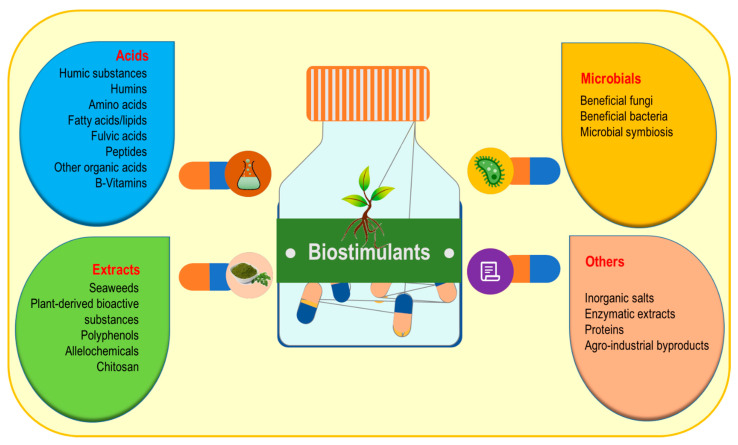
Major categories of biostimulants.

There is no specific definition of biostimulants yet, despite their regulatory functions in plant growth and development [44,45]. Biostimulants originate from natural sources and can be effectively categorized into the following four prime groups, namely, acids, microbes, plant-derived bioactive substances, and others (Figure 3) [11,46,47].

### 3.1. Microbial Biostimulants

**Figure 4:**
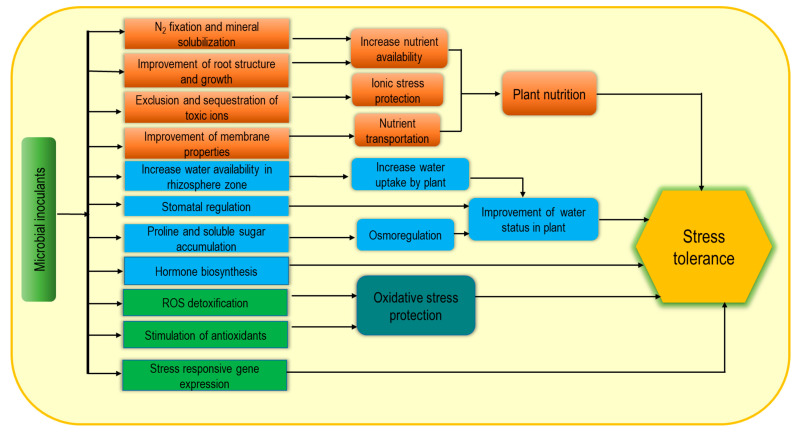
Microbial biostimulant-induced mechanism for increasing abiotic stress tolerance.

Nowadays, microbial inoculants are widely used as biostimulants due to their potential contribution as sustainable, green agricultural approaches. Plant growth-promoting rhizobacteria (PGPR), arbuscular mycorrhizal fungi (AMF), and *Trichoderma* spp. are the most common examples of microbial inoculants [44,48,49]. These may consist of only a single strain such Figureas *Bacillus subtilis* or a mixture of microorganisms displaying either additive or synergistic effects. Microbial biostimulant-induced plant growth enhancement through the improvement of biological N_2_ fixation, solubilization of minerals and other nutrients and increasing plant access to soil nutrients help to reduce the yield gap [50] under adverse environmental conditions. In addition, microbial inoculants produce volatile organic compounds (VOCs) and enhance stress tolerance, while PGPR can improve plant abiotic stress tolerance by modulating different physiological processes (Figure 4) [51,52]. Plant-microbe association-induced cell wall modification and increasing soluble sugar content are notable for enhancing water retention capacity as well as increasing plant tolerance to osmotic and ionic stress. Consequently, enrichment with exopolysaccharides (EPS) and lipopolysaccharide-protein (LP) causes cell wall modification, while a protective biofilm on the root surface is formed from polysaccharide–lipids (PL) thus resulting in higher resistance under extreme environmental stresses [53]. Moreover, PGPR also induce the biosynthesis of plant hormones including auxins, ethylene, gibberellins, cytokinins, and ABA and thus contribute to stimulating growth, nutrient uptake, delayed leaf senescence, fruit and flower formation, seed maturation, and dormancy regulation [50,54,55]. Bacteria-induced hormonal induction and EPS-mediated hydration film in roots are closely associated with increasing abiotic stress tolerance including salt, drought, extreme temperature, and pH [56]. Inoculation with PGPR (*Pseudomonas putida* strain AKMP7) was found very effective for thermotolerance through reducing ROS generation and membrane damage along with regulation of antioxidant responses including SOD, CAT and APX activities [57]. In addition, improvement of cellular metabolite accumulation including proline (Pro), soluble sugars, starch, proteins, amino acids, and chlorophyll (chl) were also observed following this inoculation as a stress tolerance mechanism. Similarly, cold-stressed plants also recovered following PGPR application through ROS scavenging, membrane stability, and enhancing stress-responsive gene expression [56,58].

Microbial inoculants are directly applied in the rhizosphere to make the association with plant roots, while seed treatment has also been documented. However, the innoculants are applied, colonization by *Trichoderma* spp., and Sebacinales (*Piriformospora indica*) in roots can transfer nutrients to the host plants. Bacterial niches spread from soil to the cell interior, their association can be temporary or permanent, and some are able to transmit from seed to the aerial parts [46]. Therefore, they positively influence the nutrient supply and use efficiency, and modulate morphogenesis by involving plant growth regulators which enhance abiotic stress tolerance. In summary, the positive effects of microbial biostimulants for enhancing abiotic stress tolerance are possibly due to their direct effect on N fixation and mineral solubilization, root growth and improvement of water availability, and the production of metabolites and hormones as well as upregulation of enzymatic activities, which are involved in enhancing plant nutrition homeostasis, osmotic adjustment and ROS regulation.

### 3.2. Acids

**Figure 5:**
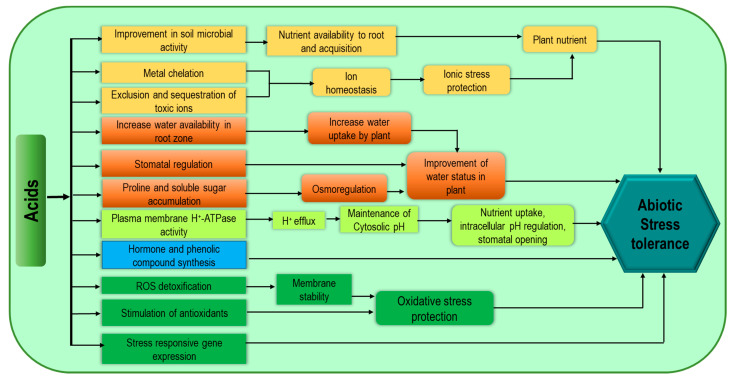
Acid-related biostimulant-mediated mechanisms for increasing abiotic stress tolerance of plants.

Humic substances (humic acid, HA; fulvic acid, FA; humins), amino acids, fatty acids, and organic acids are considered members of this group of biostimulants. Humic substances are mainly naturally produced soil organic matter not only from the decomposition process but also from microbial activity [59]. Humic substances enhance plant growth and stress tolerance through better uptake of water and nutrients [60]. Although it is unclear how humic substances influence plant physiology, their bioactivity is strongly related to the properties of the medium [61]. These substances are effective to increase plant abiotic stress tolerance as they improve water status, antioxidant capacity as well as endogenous cytokinin [56]. Humic substances cause lower accumulation of toxic Na, along with higher accumulation of N, K, Ca, Mg, P, Fe, S, Mn, and Cu which are associated with imparting better salt tolerance [62]. The modulation of ion homeostasis and the increase in Pro content with a reduction in membrane leakage (as an indicator of better salt adaptation strategy) are also attributed to humic substances [63]. Protein hydrolysate (PH) is a mixture of amino acids, peptides, polypeptides, and denatured proteins produced by both plants and animals through enzymatic, chemical, and thermal hydrolysis [48,64]. These products are available in granular or powder form and also as liquid extracts and can be applied as foliar spray or root application [48]. Protein hydrolysate products are generally recognized as agents for improving plant tolerance to environmental stresses [60]. This PH application improves soil microbial activity and biomass accumulation as well as soil respiration resulting in easy utilization of amino acids and peptides for C and N [65]. Enzymatic activities involved in carbon metabolism and nitrate assimilation are upregulated through PH supplementation as well as through enhanced plant nutrient assimilation [66]. These are well documented for their beneficial effects on enhancing water status, Pro content, phenolic compound accumulation, stomatal conductance, and photosynthetic efficiency. Stress-responsive genes also contribute to improved tolerance [67]. Moreover, PH also enhances antioxidant capacity, ROS scavenging properties and metal chelation [68]. The mechanisms associated with increasing plant stress tolerance are presented in Figure 5. Acids mediate direct improvement in soil structure through aggregate stability, better microbial activity, metal chelation, better plant nutrient uptake (especially in poor organic matter-containing soil), as well as metabolic changes including phenolic compounds, Pro synthesis, and nitrate metabolism associated with plant stress tolerance.

### 3.3. Extract-Type Products

**Figure 6:**
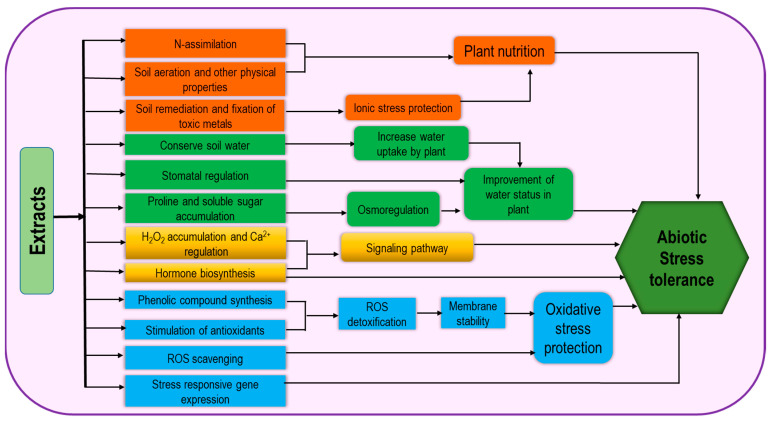
Extract-type biostimulant-induced mechanism for increasing abiotic stress tolerance.

This is a vast group consisting of different kinds of products originating from different organisms including seaweed, chitosan, plant-derived bioactive substances, polyphenols, and allelochemicals. As a source of organic matter and fertilizers, seaweeds have been long used but recently they have been recognized as effective biostimulants. Seaweed extracts (SWE) contain numerous active minerals and organic compounds which are effective in promoting plant growth, photosynthetic activity, and abiotic stress tolerance (Figure 6) and have diverse application methods including root zone application (on soils and hydroponic solutions) and foliar treatments. Therefore, they contribute to gel formation, water retention and soil aeration, heavy metal fixation, and soil remediation [46]. Regulation of stress tolerance by SWE involves the activities of antioxidants and endogenous stress-responsive gene expression [44]. Application of SWE as foliar spray causes the stimulation in nitrogen assimilation, for example, nitrate reductase activity was increased in brinjal and creeping bentgrass [50,69]. Therefore, it can be suggested that SWE could be beneficial in plant growth development by improving plant nutrition. These extracts stimulate the faster plant recovery from abiotic stress through higher membrane stability, ROS scavenging by their cofactor role in antioxidant activity and thus improve oxidative stress tolerance [67]. In addition, the role of SWE as osmoprotectants has been proven through the improvement in Pro and total soluble sugar contents under freezing stress [70]. SWE-based drought tolerance has also been demonstrated through the higher phenolics content and Pro synthesis [71].

Plant-extracted substances are not only used as food ingredients but also have the potential to be used in plant protection [72]. Although their pesticidal properties are well known, some researchers disclosed their potentiality to be used as biostimulants [73,74]. Moreover, there is a natural approach to mediate plant interactions in ecosystems through plant-extracted active compounds known as allelochemicals, which could also be an effective tool for sustainable crop management. Much attention is required for allelochemicals to be developed as biostimulant for using as cover crops and mulch crops. Research is also required to show how these approaches can be incorporated with different cropping patterns including mixed cropping, crop rotation, intercropping, etc. [46].

The deacetylated biopolymer form of chitin is chitosan, which is not widely available in nature and mostly is industrially produced through a deacetylation process from shells of crabs and shrimp, squid pens and filamentous fungi [75,76,77,78]. Their variable poly- and oligomers forms have various uses in the food, cosmetic, medical as well as agricultural sectors. Polycationic compound of chitosan can bind various cellular components like cell wall constituents, plasma membrane and even DNA, acting as plant defense elicitors due to binding with specific receptors for defense gene activation [79,80]. Therefore, it seems that chitosan and chitin exploit distinguished receptors and signaling pathways [46]. As a consequence, chitosan causes cellular H_2_O_2_ accumulation and Ca^2+^ leakage, thus playing an active role in signaling responses [81,82]. Chitosan has long been applied for plant protection against fungal pathogens, but it may also be effective for increasing abiotic stress tolerance against stressors such as salinity, drought and cold, which is supported by chitosan-mediated stomatal closure through an ABA-dependent mechanism for contributing to environmental stress tolerance [83].

### 3.4. Other Biostimulants

Beneficial elements such as Al, Si, Na, Se, Co which are not required by all plant species but are essential for particular plants for their growth promotion, are present in plants and soils as inorganic salts like chlorides, carbonates, silicates, phosphates, and phosphites [47,84]. Beneficial element-induced physiological effects have been reported which contributed to attaining plant abiotic stress tolerance including osmoregulation, cell wall rigidification, thermal regulation, plant nutrition, regulation of antioxidant responses, biosynthesis of plant hormones, and metal detoxification [84]. These inorganic salts effectively regulate stress responses by influencing hormonal signaling, osmotic status, redox homeostasis, pH, and enzymatic activity as in the case of peroxidases [46]. Thus, they deserve more attention to be used as biostimulants despite their established fertilizer uses.

There are also agro-industrial biostimulants which are basically extracts produced from food waste, industrial waste, manure, composts, vermicomposts, aquaculture residues and sewage [85]. It was reported that agro-industrial byproducts effectively improved secondary metabolite biosynthesis, phenylalanine ammonia lyase (PAL) activity, and increased crop productivity [86]. Several researchers have supported this biostimulant-mediated PAL activity along with respective regulation of gene expression [46,51,87]. Extracts of vermicompost play an active role in amplifying enzymatic antioxidants activity as well as scavenging extra ROS upon salt and drought stress [88]. However, their mode of action is yet to be accurately described due to the variation in their source materials and extraction technologies [11].

## 4. Biostimulants for the Regulation of ROS under Abiotic Stresses

### 4.1. Drought

Drought is the major yield-limiting stress factor, and it will remain so due to increased water demands of crop land driven by evapotranspiration increases related to climate change [89,90]. Drought or water deficit conditions stimulate an oxidative burst in the cells [27]. Drought-induced stomatal closure leads to photorespiration which accounts for up to 70% of H_2_O_2_ generated in the leaves [91]. However, it is not completely harmful; crop performance requires a delicate balance between ROS generation and its detoxification [24]. In many crops, water deficit occurs transiently and exogenous application of biostimulants is widely used for enhancing crop performance under different abiotic stresses [60,67]. For instance, HA application increased antioxidant enzymatic protection and improved the expression of tonoplast intrinsic proteins (OsTIP), a sub-family of aquaporins which assist in the movement of water, small uncharged solutes, and gases, which contributed to drought tolerance [88,92]. Humic acid is a product of the biodegradation of plant parts and microbes and has direct effects on plant growth and metabolism. Its growth promotive effects are reported in many crops, which cannot be attributed solely to hormone-like activity [93]. Humic acid is also found to minimize oxidative stress in plants. Its application on millet seedlings which were subjected to water stress by withholding irrigation at the three–five leaf stage, increased seedling growth and antioxidant properties. In the study, water stress increased levels of O_2_^•−^ and H_2_O_2_ in the leaves of millet; however, HA application reduced the production rate of oxygen radicals along with the decreased activity of SOD and POD. Without the regulation of antioxidant enzymes, HA showed its effect on stomatal conductance, photosystem I and photosystem II activity which improved the photosynthetic performance while also helping to decrease ROS production and maintain membrane stability [94]. The effect of PGPB and HA was examined in drought-affected sugarcane. In sugarcane, HA helped plants to recover from drought stress by enhancing the activity of SOD, CAT, and APX. On the other hand, PGPB-induced osmoregulation contributed to regulate leaf water potential and RWC by closing stomata efficiently, resulting in plant water preservation. These are involved in maintenance of the cellular microenvironment for continuing the metabolic and physiological activities in a better way, so that ROS production and oxidative stress are decreased [95]. Another biostimulant, seaweed extracts, are widely used in many crops for increasing crop production [96,97]. Application of commercial extract of *Ascophyllum nodosum* on soybean exhibited higher water content, reduced wilting and better recovery as well as improved ROS scavenging under drought conditions [98]. Similarly, the application of *A. nodosum* seaweed extracts also increased antioxidant activity and reduced lipid peroxidation in *Paspalum vaginatum* grass during water stress [71]. Regulation of Pro, protein and carotenoid contents, and activity of CAT, APX, guaiacol peroxidase, and GR activity were conferred by foliar application of beeswax waste and licorice extract which were directly involved in ROS scavenging, reduction in malondialdehyde (MDA) level and prevention of chl breakdown. In addition, improvement in the quantum efficiency of photosystem II (^Fv^/_Fm_), net photosynthetic rate, stomatal conductance, transpiration, and water use efficiency were responsible for a reduction in ROS production [99]. In field-grown maize, HA application with S-containing soil amendment, significantly increased SOD and CAT activities and reduces and H_2_O_2_ content under water stress conditions [100]. Soil microbial community also improves above-ground plant health under stress and the use of PGPR has been gaining importance as a drought management strategy [101]. Microbial inoculum of *P. fluorescens* and *Bacillus amyloliquefaciens* were reported to increase antioxidant scavenging in peppermint [102]. Thus, growing evidence suggests that the application of biostimulants may be a cost-effective strategy to overcome drought-induced oxidative stress and improve crop health under stress. Some examples of the application of biostimulants and their effects on plant ROS generation and antioxidant system are summarized in Table 1.

### 4.2. Salinity

Salt tolerance of plants is conferred by retention and/or acquisition of water, maintenance of ion homeostasis, protection of chloroplast functions, biosynthesis of osmotically active metabolites and specific proteins. Upregulation of antioxidant defense systems and ROS scavenging, protection against membrane damage, and maintenance of structural integrity of ultrastructural organelles are also vital to achieve salt tolerance [105,106]. Exogenous use of diverse products is being introduced for developing salt tolerance in plants. Table 2 summarizes the role of different biostimulants in conferring salt tolerance in plants.

Salt-affected wheat plants exhibited decreased tissue water status, disrupted ionic and hormonal homeostasis, and photosynthetic performance and some other physiological disorders. Exogenous GSH (1 mM) and *Moringa oleifera* leaf extracts (MLE, 3%) in salt-treated wheat plants increased endogenous GSH and AsA levels, attributed osmotic tolerance, stabilized membrane properties and decreased electrolyte leakage (EL). Improved tissue water status and ionic and nutrient homeostasis were maintained by GSH and MLE, additionally that also reduced ROS generation [107]. *Catharanthus roseus* treated with 150 mM NaCl showed physiological disarray and oxidative damage. Spraying with chitosan nanoparticles (CSNPs, 1%) resulted in alkaloid accumulation, impeded chl breakdown and upregulated activities of CAT, APX, and GR. Consequently, CSNPs lessened the oxidative damage, evidenced by decreased MDA and H_2_O_2_, thus allowing smooth membrane activity and ensuring salt tolerance [108]. Ait-El-Mokhtar et al. [109] investigated the role of exogenously applied AMF and/or compost (240 mM) in regulating oxidative damage in date palm (*Phoenix dactylifera* cv. Boufeggous) under salinity (240 mM NaCl). Salt stress caused a higher accumulation of Na^+^ and Cl^−^, disrupted osmotic adjustment and antioxidant system. As a result, H_2_O_2_ content and lipid peroxidation level increased significantly, compared with control. Regulation of Pro and soluble sugar helped to stabilize the membrane to a great extent which decreased membrane lipid peroxidation. The AMF- and/or compost-treated plants showed increased SOD, APX, and CAT activities which are correlated with the decreased H_2_O_2_ content and membrane lipid peroxidation. Improved stomatal conductance, leaf water potential, content of soluble sugar, K, and Ca together with decreased Na and Cl content also resulted from AMF and/or compost addition under drought stress. These results prove the pivotal role of AMF and/or compost in enhancing antioxidant properties and decreasing oxidative stress [109]. Salt stress altered chl content and components of chl fluorescence which resulted in oxidative stress. Salt stress caused oxidative damage in cucumber seedlings as indicated by the increase in H_2_O_2_ and MDA levels. Exogenous 5-aminolevulinic acid (ALA) (25 mg L^−1^) addition increased AsA/DHA, GSH/GSSG, ascorbic acid oxidase (AAO), APX, MDHAR, DHAR, and GR in salt (NaCl, 50 mM)-stressed cucumber plants. Exogenous ALA augmenting the AsA/GSH pathway diminished the H_2_O_2_ scavenging system [110]. Upregulation of antioxidant system constituents AsA and GSH, the activity of APX, MDHAR, DHAR, and GR in vanillic acid (40 and 50 μM)-treated, salt-affected tomato plants reduced ROS generation, decreased LOX activity and membrane injury. The VA-treated plants also showed higher photosynthetic pigment levels [111]. Improved physiology in salt-affected plants contributed by various biostimulants is one of the prerequisites which imparts reduced ROS production. Again, upregulated antioxidant defense system (contributed by biostimulant supplementation) is directly involved in ROS scavenging and oxidative stress reduction in plants under salinity.

**Table 2:** Role of biostimulants in regulating antioxidant defense and ROS under salt stress.

Crop Species	Salinity Levels and Duration	Biostimulant Type and Dose	Antioxidant Defense and ROS Regulatory Effects	Reference
*Triticum aestivum* L. cv. Sakha 93	9.10 dS m^−1^ NaCl; 30 d after sowing (DAS) to 50 DAS	Fresh MLE (3%) and GSH (1 mM)	Increased endogenous GSH and AsA levels. Stabilized membrane integrity Decreased EL. Prevented chl breakdown.	[107]
*Vigna unguiculata*	Seawater, 3.5 and 7 dS m^−1^; vegetative stage	*Foeniculum vulgare* (FSE) and *Ammi* seed extracts	Decreased EL, MDA, H_2_O_2_, and O_2_^•−^ Improved membrane stability index (MSI).	[112]
*Dracocephalum moldavica* L.	50–100 mM NaCl	Fe_2_O_3_ nanoparticle; 30, 60, and 90 ppm	Increased total phenolic, flavonoid and anthocyanin contents. Improved the activities of guaiacol peroxidase, APX, CAT and GR.	[113]
*Cucumis sativus* L.	50 mmol L^−1^ NaCl, at vegetative stage	ALA, 25 mg L^−1^	Decreased H_2_O_2_ and MDA levels Increased AsA/DHA, GSH/GSSG. Increased ascorbic acid oxidase (AAO), APX, MDHAR and DHAR activity. Augmenting the AsA/GSH pathway exogenous ALA diminished the H_2_O_2_ level.	[110]
*Solanum lycopersicum* L. cv. Pusa Ruby	150 mM NaCl; at 10-d-old seedlings for 5 d	Vanillic acid (40 and 50 μM	Upregulation of AsA and GSH level. Improvement of APX, MDHAR, DHAR and GR activity. Downregulated ROS generation. Decreased LOX activity and membrane injury.	[111]
*Brassica napus* L.	1.5 dS m^−1^, 5 dS m^−1^ and 10 dS m^−1^ NaCl; throughout the growing period	Ca-fortified composted animal manure (Ca-FCM; 1, 2 and 3%)	Modulation of SOD, APX, CAT, GPX, GR and GST activities. Decreased EL and chl breakdown.	[114]
*Chenopodium quinoa*	Saline soil, 20 dS m^−1^, throughout the growing period	*Burkholderia phytofirmans* PsJN (CFU = 109) and biochar (1% *w*/*w*)	MDA and O_2_^•−^ content decreased. Modulated SOD, APX, GR, GPX and GST activity. Modulated the GSH, GSSG and GSH/GSSG. Improvement of relative membrane permeability and membrane stability index.	[115]
*Catharanthus roseus*	150 mM NaCl, vegetative stage	Chitosan nanoparticles (CSNPs, 1%)	Impeded chl diminution. Stimulated CAT, APX and GR activity Lessened MDA level and H_2_O_2_ production.	[108]
*Arachis hypogaea* L.	2.5, 5, 7.5, 10, 12.5, and 15% NaCl; 72 h	Endophytes like *Bacillus firmus* J22N and *Bacillus* sp. REN51N	Increased activity of SOD, GR, CAT and APX. Decreased H_2_O_2_.	[116]
*Phoenix dactylifera* cv. Boufeggous	240 mM NaCl; 5 months after germination, 2 weeks	AMF and/or compost	Pro and soluble sugar regulation. Improved SOD, APX and CAT activities. Reduced H_2_O_2_ content and lipid peroxidation. Checked chl degradation.	[109]
*Vigna radiata*	150 mM NaCl; After 5 d of spore suspension application NaCl was added up to 35 d	*Aspergillus awamori* (EWF)	Pro, polyphenols, flavonoids and tannin accumulation increased. CAT and APX activity enhanced. Lipid peroxidation reduced.	[117]

### 4.3. High Temperature

Temperature rise beyond the tolerance level causes severe stress in plants which directly affects plant functioning [118]. However, to check the physiological and biochemical decay due to oxidative stress under high temperature (HT), plants develop different tolerance mechanisms which include protective solutes formation, enzyme activation and gene expressions [119]. Multiple biostimulants studied under different groups have been applied exogenously in achieving plant tolerance to HT (Table 3). However, HT often occurs with water deficit stress and osmotic stress thus making it complex in nature to determine the underlying mechanism.

Endophytic bacteria can increase plant growth and improve crop production by diminishing the negative effects of HTs. *Bacillus cereus* SA1 mitigated the HT (40 °C; up to 10 days) effect in soybean by reducing MDA accumulation which was comparatively higher in only stressed conditions [120]. This might be due to the higher antioxidant activity in inoculated plants where APX activity increased fourfold compared to the untreated stressed plants. This plant growth-promoting endophytic bacteria (PGPEB; *B. cereus* SA1) had been used in combination with HA on 37 °C-stressed tomato seedlings [121]. Thereafter, HT-induced higher ABA and lower SA and amino acid content had been reversed in SA1+HA-treated stressed seedlings, while SA1 + HA treatment also caused the higher response of antioxidants including SOD, APX, and GSH as well as better plant nutrition which resulted in a 98% reduction in MDA level. In contrast, a study with thermotolerant plant growth-promoting strain *Pseudomonas putida* AKMP7 reduced the oxidative damage by reducing antioxidant enzymes activity and reducing the ROS generation in HT stressed wheat seedlings at 37–40 °C. Inoculation significantly reduced MDA content and the activity of SOD, APX, and CAT under HTs [58]. Sarkar et al. [122] primed two wheat cultivars with *B. safensis* and *Ochrobactrum pseudogrignonense* and observed the oxidative responses and ROS detoxification under HTs (40 °C) for different time periods. Results showed that ROS generation such as H_2_O_2_ and O_2_^•**–**^ significantly increased over time. In contrast, H_2_O_2_ level reduced by 30% and 44% under 12 h HTs condition due to application of *B. safensis* and *O. pseudogrignonense,* respectively. In contrast, higher O_2_^•**–**^ level was observed at 40 °C which was later reduced by priming with PGPRs, especially *B. safensis*. Moreover, antioxidant enzymatic activity (APX, SOD, POX, and GR) increased while reducing MDA content and EL due to PGPRs treatment under HTs. Earlier, different PGPBs were used in chickpea, sorghum, and wheat and resulted in less oxidative damage and much antioxidant enzymes activities, as a sign of heat tolerance mechanism [123,124,125]. Beside the above-mentioned protective roles of PGPRs, their ability to increase photosynthetic rate and nutrient uptake, production of phytohormones are also considerable mechanisms which facilitate plants to survive in HT condition [121].

Arbuscular mycorrhizal fungi are omnipresent soil microbes that play a vital role in nutrient mineralization and improving water availability in soil root regions. Duc et al. [126] used AMF to mitigate HT effects in tomato plants. Results showed that, both MDA and H_2_O_2_ contents increased under HTs (42 °C) which were later reduced by about 40% due to application of AMF, compared with control plants. Furthermore, they observed enhanced antioxidant enzymatic activity in both roots and leaves. Although POD activity increased by 70% when treated with *S. constrictum* under drought stress, SOD activity nearly doubled under HTs with the same inoculant compared with control. In addition, Kumar et al. [127] observed biochar effects under HTs in *Arabidopsis.* Results revealed that, under HTs (50 °C), lipid peroxidation significantly increased compared with control samples. However, biochar protected plants from lipid peroxidation caused by HTs by significantly reducing MDA content. The hyphae of the AMF can colonize and branch extensively, facilitating water and nutrient uptake for plants and prevent the photosynthetic apparatus from HT-induced damage which is very important regarding stress tolerance [128].

Seaweed extracts (SWE) derived by extracting several macroalgae species are now widely used substances having the potentiality to reduce adversity of abiotic stress, thus enhancing plant productivity [129]. Anjos Neto et al. [130] experimented with five concentrations of *A. nodosum* SWE in spinach seedlings under HT (30 °C) and observed that in both non-stressed and HT-stressed conditions, MDA and H_2_O_2_ contents were reduced due to the application of 0.30% SWE. Furthermore, improved activity of antioxidant enzymes were also observed with SWE which could have reduced the oxidative degradation by lower MDA content and cell oxidative damage under HTs. The ability of SWE to provide a good level of seed vigor facilitates plants with a better initial growth which can also be considered as a protective mechanism against HT stress.

### 4.4. Low Temperature

Different experimental findings have reported the positive role of different biostimulants under low temperature (LT) stress in different crop species (Table 3). AMF inoculation increases mineral nutrition, water status and secondary metabolites production in plants. Pasbani et al. [131] studied cold stress mitigation in eggplants by colonization with AMF. Four AMF species were applied at 5 °C for one week and observed that AMF bring cold stress tolerance to plant by improving the photosynthetic parameters and activating the antioxidant defense mechanisms. Both leaf concentrations of MDA, H_2_O_2_, and EL were increased at 5 °C stressed conditions. Moreover, the enzymatic antioxidant capacity of APX, SOD, and CAT were significantly increased with mycorrhization treatment compared with controls under LTs. Chu et al. [132] also reported that EL, lipid peroxidation, O_2_^•−^ production, and H_2_O_2_ content increased under 5 °C for 5 days in both the cultivars (Zhengdao and Kangma) of *Elymus nutans* Griseb. However, *Glomus mosseae* reduced superoxide in both cultivars by 37 and by 16%, respectively, compared to control. However, inoculation with AMF also benefits plants with increased water and nutrient uptake, higher CO_2_ fixation, enhanced accumulation of phenolics, sugar, and proline etc., which are also helpful against LT stress [131].

Soil inoculated with PGPRs has been reported with increasing abiotic stress tolerance in plants. For instance, *Rhizobium* under different abiotic stress conditions successfully enhanced plant physiological growth [133]. A thermotolerant bacterial biostimulant namely, *P. putida* strain AKMP7, reportedly increased root-shoot length and biomass under HTs. Experimental findings also suggest that this bacterial strain alleviates oxidative stress damage by lowering ROS generation by upregulating antioxidant enzyme SOD, CAT, and APX activity in wheat seedlings [57]. Subramanian et al. [58] experimented with psychrotolerant soil bacteria to alleviate LT stress in tomato plants. Results showed that the bacterial strains substantially enhanced the antioxidant defense system under LT stress conditions at 15 °C. Notable plant tolerance to chilling stress through reduced MDA content and activation of antioxidant enzymes (SOD, APX, and GR) has been observed. The activity of APX and GR was observed to be notably higher in plants treated with *P. vancouverensis* OB155 than in control plants. In addition, GSH content and Pro synthesis in the leaves were also increased by bacterial strains treatments, compared with the respective controls.

Yuan et al. [134] experimented to reveal the positive effect of biochar on rice seedlings under cold stress at 10 °C for 3 weeks. The findings suggested that MDA content increased under cold stress condition which was however reduced by 30% with 10% biochar application. On the contrary, at lower biochar concentrations, H_2_O_2_ increased but immediately reduced by 45% at higher concentrations. In addition, three primary antioxidant enzymes (SOD, POD, and CAT) activities that protect the plant from cold stress were increased when biochar was applied. An increase in the content of soluble sugar, Pro, with increasing biochar treatment is a clear indication of biochar’s role against cold-induced oxidative damages.

Pokluda et al. [135] worked with two biostimulants named Asahi SL or Goëmar Goteo (Arysta Life Science) on coriander (*Coriandrum sativum* L.) leaf extract under chilling stress at 6 °C. Results revealed that EL significantly declined in chill-stressed plants due to the application of biostimulants. Although stress indicators such as lipid peroxidation and H_2_O_2_ concentration reduction rate did not confirm the protective role of the biostimulant at chilling temperature, it enhanced the photosynthetic capacity of photosystem II, transpiration, and stomatal conductance to its maximum. Besides this, total phenolic compounds, L-ascorbic acid content, and total antioxidant activity were increased due to biostimulant application under chilling stress.

Different seaweed extracts could be another promising approach to mitigate LT stress in plants. Bradacova et al. [136] investigated the role of different seaweed extracts, and rhizobacteria with PGPRs to improve the low root zone temperature tolerance in maize. At 12–14 °C root zone temperature for two weeks, they observed that application of Algafect (extracts from *A. nodosum, Fucus* spp., *Laminaria* spp.) at 16 mg kg^−1^ resulted in decreased leaf damage, increased shoot and root growth, and increased root length density of maize plants. This finding confirms that SWE are associated with increasing SOD activity in the root and leaf tissue with key functions in the antioxidant defense system.

**Table 3:** Protective role of biostimulants in plant under high and low temperature stress.

Crop Species	Level of Stress and Duration	Biostimulants and Dose	Beneficial Effects	Reference
*Glycine max* L.	35 °C, 2 h each for 2 d	FA, 2.0 mg L^−1^	Increased RWC and activity of SOD, APX and GST. Reduced oxidative damage, H_2_O_2_ and MDA content.	[137]
*Spinacia oleracea*	30 °C, 6 h	SWE, 0.15, 0.30, 0.60 and 1.2%	Reduced MDA and H_2_O_2_ contents.	[130]
*Triticum aestivum*	40 °C, 12 h	PGPRs strains of *Ochrobactrum pseudogrignonense* and *Bacillus safensis*	Improved cell viability, SOD, POX, CAT, APX and GR activity. Reduced EL, H_2_O_2_, O_2_^•−^ and membrane damage.	[122]
*Triticum aestivum*	37–40 °C, 95 d	PGPRs strains (*Pseudomonas putida*; AKMP7)	Reduced membrane damage and ROS generation. Increased SOD, APX and CAT activity. Improved Pro and sugar content.	[57]
*Lycopersicon esculentum* Mill.	38 °C, 7 d	PGPRs strains (*Agrobacterium tumefaciens)*	Reduced EL and lipid peroxidation. Increased SOD, CAT, POD and APX activity.	[138]
*Solanum lycopersicum* L. landraces E17, E36, E107, PDVIT	Elevated temperature (up to 42 °C) for whole growing period	CycoFlow (sugarcane molasses with yeast extract), 400 mL plant^−1^	Increased the content of reduced AsA and total AsA. Reduced the hydrophilic antioxidant activity and enhanced the lipophilic antioxidant activity.	[139]
*Coriandrum sativum* L.	6 °C, 6 d	Asahi SL (synthetic) and Goëmar Goteo (*Agrobacterium nodosum*) as 0.1%, foliar spray	Reduced the content of MDA and H_2_O_2_ content as well as the EL. Increased total antioxidant activity, total phenolic content.	[135]
*Oryza sativa* L.	10 °C, 21 d	Biochar, 1, 3, 5, 7 and 10%	Increased soluble sugar content, antioxidant activity, SOD and POD activity. Reduced lipid peroxidation.	[134]
*Solanum melongena* L. cv. Yalda	5 °C, 7 d	AMF (*Funneliformis mosseae*, *Claroideoglomus etunicatum*, *Rhizophagus irregularis*, and *Diversispora versiformis*)	Enhanced SOD, CAT, APX, PAL and POD activity. Increased carbohydrate, soluble sugar and free phenolics content. Reduced membrane damages, EL and H_2_O_2_ content.	[131]
*Elymus nutans*	5 °C, 5 d	AMF (*Glomas mosseae*)	Decreased oxidative damage, EL, H_2_O_2_ and O_2_^•−^. Increased SOD, CAT, APX and GR activity. Improved antioxidant components such as GSH and soluble sugar content.	[132]
*Citrullus lanatus* Thunb. cvs. Crimson Sweet and Charleston Gray	4 °C, 36 h	AMF (*Glomus intraradices*)	Lowered the EL, MDA and H_2_O_2_ contents. Enhanced POX activities.	[140]
*Lolium perenne* L.	4.2 °C (average), 10 d	AMF (*Glomas intraradices*)	Increased activities of SOD, POD and CAT. Reduced MDA content.	[141]
*Lolium perenne* L.	4.2 °C (average), 10 d	Biochar, 4%	Increased activities POD and CAT but declined SOD activity and MDA content.	[141]
*Hordeum vulgare* L. cvs. Abida and Nik	5 °C, 21 d	AMF (*Rhizophagus irregularis*)	Reduced membrane leakage, MDA and H_2_O_2_ contents. Upregulated SOD, CAT and POD activity.	[142]
*Camellia sinensis* L. O. Kuntze cv. Anji Baicha	−4 and −8 °C, 24 h	Chitosan oligosaccharide (COS) solution, 1.25 mL L^−1^	Enhanced SOD and POD activity.	[143]

### 4.5. Metal/Metalloid Toxicity

Increasing urbanization and industrialization of the modern world are resulting in excess heavy metal (HM) accumulation in soil which is a potential threat for plant survival. Exposure to different toxic metals hampers plant morpho-physiological and biochemical mechanisms which are directly or indirectly related to metal-induced oxidative stress and ROS production. Based on the process of generating ROS, HMs are categorized into two groups. Firstly, the “redox-active” ones with the ability to produce ROS through direct reactions (Haber–Weiss and Fenton) examples of which include Cu, Fe, Co, and Cr. Secondly, the “redox-inactive” ones which produce ROS indirectly with the help of different metabolic processes (e.g., replacement of enzymatic cations, NADPH oxidase activation, reduction in antioxidant GSH pool, etc.), examples of which include Cd, Ni, Pb, Al, and Zn [144]. Irrespective of direct or indirect initiation, high or toxic amounts of HMs inhibit growth and cease metabolism. So far, a number of defensive approaches inside and outside of plants have been discovered but all those fail beyond a certain limit of these toxic HMs [145]. Considering this issue, researchers worldwide are focusing more on the minimization of HM accumulation in plant cells by using external biotic or abiotic agents. Use of biostimulants is not new in that case, but with the course of time newer types of biostimulants are getting attention depending on the characteristics of the HMs studied (Table 4).

Organic acids like ethylenediaminetetraacetic acid (EDTA), γ-aminobutyric acid (GABA), maleic acid, citric acid, FA, and HA are gaining popularity among scientists and have been proven effective against HM stress. Mahmud et al. [146,147,148,149] used *Brassica juncea* L. as the test crop and exposed it to Cd stress for 3 days. When exposed to 0.5 mM EDTA, 25.6, 26.1, and 27.6% reductions in thiobarbituric acid reactive substances (TBARS), H_2_O_2_ content, and LOX activity, respectively, were observed in Cd (1.0 mM)-stressed seedlings compared with seedlings receiving only Cd treatment. The supplementation of EDTA increased the GSH levels by 11% in both levels (0.5 and 1.0 mM Cd) of stress and reduced the GSSG levels by 21% and 18% under 0.5 and 1.0 mM Cd stress, respectively, compared with Cd stress alone. Upregulation of antioxidant enzyme (APX, DHAR, MDHAR, GR, SOD, CAT, and GPX) activities was also observed due to EDTA application [149]. Two levels of CA co-treatment on mustard plants exposed to two levels of Cd stress also showed almost similar results [148]. Similarly, FA and/or HA were also reported to help in tolerating Cd toxicity [150,151]. Not exclusive to Cd, this positive phenomenon is also applicable for chromium (Cr)-stressed *B. juncea* plants treated with GABA [146] and MA [147], and wheat plants treated with FA [152]. Such organic acids are reported to induce metal stress tolerance in plants either by upregulating the antioxidant defense system, or by playing other diverse roles, for example: reducing solubility and bioavailability of toxic ions, chelating or precipitating metal ions, providing osmoprotection, reviving photosynthetic pigments etc.

The use of soil amendments is another convenient technique of protecting plants from HM stress. Different percentages of biochar were used in *Spinacia oleracea* [153] and *Brassica chinensis* L. [154] plants grown on Cd-contaminated soil and the data manifested the protective roles of biochar against Cd stress. Being a larger component with an alkaline and porous nature, biochar can absorb or stabilize toxic metals and hence reduce their availability to plants [155,156].

Plants sometimes develop an alliance with different soil microorganisms like bacteria, fungi, or algae which is advantageous in many ways including plant protection against metal toxicity. These microorganisms have the ability to survive in a wide array of environments and so various species are used to minimize HM uptake or accumulation. For example, AMF, PGPRs, Rhizobia, or other genera of microbial families are nowadays being incorporated with crop species to facilitate better tolerance to adverse factors of soil. Zhang et al. [157] selected two species of AMF, *Rhizophagus intraradices* (Ri) and *G. versiforme* (Gv), and inoculated them in the Cd-contaminated soil of maize cultivation. Their results revealed that, through the upregulation of non-enzymatic GSH and phytochelatins contents, AMF can assist plants in combating damage caused by Cd. Similarly, other two species of fungi *Mucor circinelloides* and *Trichoderma asperellum* were also reported to upregulate SOD and CAT activities and thus conferring Cd tolerance in *Arabidopsis thaliana* plants [158]. The inoculation of *T. asperellum* has also proven successful in mitigating copper (Cu) stress in onion plants, by reducing lipid peroxidation in leaves, roots, and bulbs [159]. Other important microorganisms, PGPR (*Paenibacillus mucilaginosus*) and rhizobium (*Sinorhizobium meliloti*) were chosen by Ju et al. [160] for checking the efficacy against Cu stress in *Medicago sativa* plants where a remarkable decline in the production of harmful H_2_O_2_, O_2_^•−^, and MDA, and the activities of SOD, CAT, and APX were observed. These microorganisms enhance metal tolerance through the production of chelating agents, stimulation of root growth, and development of soil microbial community and improvement of nutrient and water availability [160].

**Table 4:** Effect of different biostimulants on the regulation of ROS under metal/metalloid stress.

Crop Species	Metal/Metalloid Dose and Duration	Biostimulant Type and Dose	ROS Regulatory Effects of Biostimulants Used	Reference
*Oryza sativa* L. cv. BRRI dhan29	Cd (0.25 and 0.5 mM CdCl_2_), 3 d	Ca (2.5 mM CaCl_2_), co-treatment	MDA and H_2_O_2_ contents, and LOX activity were reduced. Increased contents of DHA and GSSG were diminished by Ca, which was vice-versa for AsA. Enhancement in MDHAR, DHAR, GR and SOD activities.	[161]
*Oryza sativa* L. cv. BRRI dhan29	Cd (0.3 mM CdCl_2_), 3 d	Mn (0.3 mM MnSO_4_), co-treatment	MDA, H_2_O_2_ contents and LOX activity were reduced. Increased AsA and decreased DHA contents. Increased DHAR and CAT activities. Enhanced SOD and MDHAR activities.	[162]
*Brassica juncea* L. cv. BARI Sharisha-11	Cd stress (0.5 and 1.0 mM CdCl_2_), 3 d	Citric acid (0.5 and 1.0 mM), co-treatment	MDA, H_2_O_2_ contents and LOX activity decreased. AsA and GSH contents increased but DHA and GSSG contents decreased. SOD, CAT, DHAR, MDHAR, and GR activities upregulated.	[148]
*Brassica. juncea* L. cv. BARI Sharisha-11	Cd stress (0.5 and 1.0 mM CdCl_2_), 3 d	EDTA (0.5 mM), co-treatment	26, 26, and 28% reduction in TBARS, H_2_O_2_ contents and LOX activity, respectively in 1.0 mM Cd-stressed seedlings compared to Cd-stressed seedlings alone. AsA content was restored but DHA and GSSG contents reduced, while GSH level further increased. AsA-GSH pathway enzyme activities increased along with SOD, CAT and GPX activities.	[149]
*Lactuca sativa* L.	Cd (20 μM), 14 d	FA (0.5 g L^−1^), foliar application	EL, MDA, H_2_O_2_ and O_2_^•−^ contents were reduced. Reduced SOD and POD activities and increased CAT and APX activities.	[150]
*Lepidium sativum* cv. Helen	Cd (100 and 200 mg kg^−1^ soil)	HA + FA (3500, 5250 and 7000 mg L^−1^), soil drenching	Minimized MDA and H_2_O_2_ contents. Differential changes in the data of CAT, POD and SOD activities were reported.	[151]
*Brassica chinensis* L.	Cd (5 and 10 mg kg^−1^ soil), 30 d	Biochar (2.5 and 5%)	Efficient reduction in MDA and H_2_O_2_ contents were documented. GSH content and POD, SOD, APX, CAT activities increased while GR activity was decreased.	[154]
*Spinacia* *oleracea*	Cd (25, 50 and 100 mg kg^−1^ soil), 52 d	Biochar (3 and 5%)	The contents of MDA and AsA were reduced.	[153]
*Arabidopsis thaliana*	Cd (10, 50, 100 mg kg^−1^ soil) or Pb (100, 500, 1000 mg kg^−1^ soil), 35 d	*Mucor circinelloides* (MC) or *Trichoderma asperellum* (TA)	Increased activities of SOD and CAT.	[158]
*Zea mays*	Cd (1 or 5 mg kg^−1^ soil), 70 d	AMF (*Rhizophagus intraradices* and *Glomas versiforme*) (5%)	Induced higher GSH and phytochelatins production.	[157]
*Brassica juncea* L. cv. BARI Sharisha-11	Cr (0.15 and 0.3 mM K_2_CrO_4_), 5 d	GABA (125 μM), co-treatment	Reductions in MDA, H_2_O_2_ contents and LOX activity were observed. AsA and GSH contents increased but DHA and GSSG contents decreased. Activities of antioxidant enzymes measured were upregulated, except for APX at severe stress.	[159]
*Brassica juncea* L. cv. BARI Sharisha-11	Cr (0.15 and 0.3 mM K_2_CrO_4_), 5 d	Maleic acid (0.25 mM), co-treatment	MDA, H_2_O_2_ contents and LOX activity were reduced. AsA and GSH contents increased but DHA and GSSG contents decreased. Activities of antioxidative enzymes measured were upregulated.	[147]
*Triticum aestivum* cv. Lasani 2008	Cr (0.25 and 0.5 mM K_2_Cr_2_O_7_), 90 d	FA (1.5 mg L^−1^), foliar spray	Upregulation of CAT and APX activities in both shoot and root were observed.	[152]
*Oryza sativa* L. cv. BRRI dhan29	As (0.5 and 1 mM Na_2_HAsO_4_), 5 d	Ca (10 mM CaCl_2_), co-treatment	MDA and H_2_O_2_ contents decreased by 27 and 13%, respectively by Ca supplementation in 1 mM As-stressed seedlings. Modulated AsA, DHA, GSH and GSSG level. Activities of SOD, CAT, APX and MDHAR increased.	[163]
*Medicago sativa*	Cu contaminated soil, 90 d	*Paenibacillu smucilaginosus* and *Sinorhizobium meliloti* co-inoculation	Reduced the MDA, H_2_O_2_ and O_2_^•−^ contents. Lower SOD, CAT and APX activities were recorded.	[160]
*Allium cepa* L.	Cu (50, 100 or 250 µM CuSO_4_·5H_2_O), 8 d	*Trichoderma asperellum* inoculation	Decreased MDA content.	[159]

### 4.6. Waterlogging/Flooding

Flooding or waterlogging (WL) affects crop survival in areas with frequent events of excessive rainfall, unpredictable changes in the water table and improper drainage [164]. Oxygen availability is crucial for plant metabolism and growth, but water in excess reduces this availability of oxygen in plant cells. This results in morphological, physiological and metabolic disturbances, including inhibition of shoot and root growth, water and nutrient uptake, photosynthesis, root respiration etc. Like other environmental stresses, flooding or WL accelerates excess ROS generation which ultimately invokes oxidative stress in plants. Worldwide many researchers have experimented with different types of protectants or practices to find out the best possible ways to minimize WL-induced damages. Using various biostimulants is one of those. The literature available are scarce, but most of these available studies have proven the positive effects of biostimulants against WL stress (Table 5).

An et al. [165] used ALA (5 mg L^−1^) in mitigating WL stress in fig (*Ficus carica*) seedlings and reported that ALA pretreatment slowed down the O_2_^•−^ production rate by almost 62.07%, compared to the controls. In addition, reduction in MDA content and enhancement of antioxidant enzyme activities indicate that ALA pretreatment can promote antioxidant capacity and minimize membrane damage of waterlogged fig plants. Another important mechanism mentioned is the ability of ALA to stabilize root vigor and hence enhance water uptake which helps in maintaining water balance under waterlogging stress [165].

Wheat plants waterlogged for 5 d were inoculated with *T. asperellum* (strain MAP1), a fungal endophyte which was isolated from the roots of *Canna indica* L., which resulted in diminishing WL stress-induced MDA, H_2_O_2_ and EL contents and regulating antioxidant system [166]. The ability of MAP1 inoculation to produce IAA, Pro, total phenols, flavonoid, and having the potential to scavenge free radicals helped it to facilitate plants with tolerance against WL stress.

In addition to these above-mentioned studies, endogenous Zn level in seeds is also reported to give positive output in alleviating WL stress-induced damages in wheat by modulating the antioxidant defense system and regulating ROS production [167]. Yet, if compared to morphological, physiological, genomic, or proteomic responses, a very limited number of studies have been conducted on biostimulant-induced oxidative stress responses of cultivated crops under flooding or WL conditions. So, further studies focusing on WL-induced oxidative damages and relevant antioxidant defense systems are needed.

## 5. Limitations of Using Biostimulants

Due to the beneficiary roles of biostimulants, their application can cause significant increases in crop production under abiotic stresses. However, certain obstacles remain that restrict the wide use of biostimulants, especially in-field directly by farmers. Biostimulants are dose- and crop-specific, hence posing major challenges to develop appropriate biostimulant formulas and doses for field use. As the mode of action of every biostimulant is very precise, a group of biostimulants are not expected to be effective against all types of stresses offering extended cross-protection. Moreover, the efficiency of biostimulants also depends on the proper interactions among the crops, environment, and biostimulants. As PGPRs have an active role in phosphorus mobilization [50], they are able to increase the growth and yield of plants when grown in inorganic phosphate limiting soils. On the other hand, application methods are also varied according to the variation of biostimulants. Microbial inoculants must be applied at the rhizospheric zone or as seed treatment while others can be used as a foliar spray and along with nutrient solutions. Biostimulants can also become phytotoxic if applied at high doses, and there is controversy about the phytotoxicity of animal-derived PHs in particular, while commercial PHs of plant origin cause stimulation of plant growth under Fe deficient conditions [60]. Therefore, such demonstrated positive and negative impacts raise questions about the economic feasibility of the use of humic substances for increasing crop yields [11].

Categorizing and identifying the mechanisms of action of biostimulants on the basis of natural raw materials is difficult because raw materials can be affected by environmental location, growing season, crop species, particular organ, and growth phases of crops [168]. Thus, the use, regulation, registration, and certification for marketing of biostimulants requires collaboration among biologists, chemists, plant physiologists, and others from sales and distribution as well as field producers [169,170].

## 6. Conclusions and Future Perspectives

Plants growing in the field face multiple stresses simultaneously which cause complex forms of damage to the plant cells, physiology, growth and development, and ultimately lead to substantial yield loss. The generation of ROS is a common response in all stresses and understanding their signaling and damaging action will help in developing stress tolerant crop varieties. Plant scientists are working continuously to find ways to prevent the damaging effects and to manage and sustain crop yields by cost-effective methods. Such a breakthrough was achieved with the discovery of an array of biostimulants for sustaining and improving plant productivity. A clear and specific definition of biostimulants is not yet approved by any authority. Moreover, biostimulants are highly dose-dependent, and crop-specific, specific to the growth and developmental stages of the plants. The complexity and heterogeneity in the mode of action of biostimulants are creating challenges in deciphering of their interaction with antioxidants system and regulation of ROS homeostasis. Determining a prescribed perfect combination of biostimulants considering plant growing microclimate is crucial but complicated. The genetic response of plants in the above-mentioned matter is more intricate. ROS signaling function, signaling role of different biostimulants (either single or interaction of multiple biostimulants) and ROS biostimulants interaction related to stress adaptation or tolerance needs to be scrutinized further employing state-of-the-art omics platforms and modern gene-editing approaches.

## Figures and Tables

**Table 1:** Role of biostimulants in regulating antioxidant defense and ROS under drought stress.

Crop Species	Stress Type and Duration	Biostimulant Type and Dose	ROS Regulatory Effects of Biostimulants Used	Reference
*Setaria italica* (L.) Beauv.	Watering withdrawal at 3–5 leaf stage up to 10 days	HA, seed soaking (100 mg L^−1^)	Reduced the generation of O_2_^•−^ and H_2_O_2_ Decreased activity of SOD and POD.	[94]
*Saccaharum officinarum* L.	After 90 days, irrigation was withheld for 21 days (up to 13% moisture content)	HA (400 mL per 9 kg Soil)	SOD, CAT and APX activities were higher in root as well as in leaves after rehydration.	[95]
*Zea mays*	Water stressed field received only 67% water of evaporation loss (at every three days as compared to no stress field which received daily 100% water of evaporation)	1250 kg S and 37.5 kg HA ha^−1^	MDA and H_2_O_2_ content decreased Increased SOD and CAT activities with reduced POD activities.	[100]
*Glycine max*	Withholding irrigation, at 14 days after planting for 75 h	7.0 mL L^−1^ commercial extract of *Ascophyllum nodosum*	Treated plants exhibited higher free-radical scavenging activity.	[98]
*Paspalum vaginatum*	Irrigation intervals were 2 and 6 days up to 6 weeks	Foliar spray of 5- or 7 mL L^−1^ *A. nodosum* extract	Decreased DPPH antioxidant and lipid peroxidation.	[71]
*Mentha piperita*	Drought stress was imposed as 50% field capacity (mild stress, irrigation until 10 days before harvest and 35% field capacity (severe stress, irrigation until 20 days before harvest)	PGPR (*Pseudomonas fluorescens and Bacillus amyloliquefaciens*), 1 mL bacterial suspension per 250 g growing media	The activity of SOD and total peroxidase were enhanced. Lipid peroxidation decreased by 50 and 70% under mild and severe water stress, respectively. Antioxidant scavenging capacity increased by two folds (DPPH and AsA equivalents).	[102]
*Ocimum basilicum* L.	50% soil water holding capacity was maintained for the whole growing season	Foliar application of palm pollen grain extract 1.0 g L^−1^ at 30, 45 and 60 days after transplanting	Activities of SOD, CAT and guaiacol peroxidase increased. AsA and GSH contents increased.	[103]
*Zea mays* and *Glycine max*	Near to permanent wilting point (−1.5 MPa) after 10 weeks of growth	Mixture of nutrients, HA and FA (25 to 300 L ha^−1^)	SOD, CAT and APX activities increased.	[104]

**Table 5:** Effect of different biostimulants on the regulation of ROS under waterlogging stress.

Crop Species	Waterlogging Duration	Biostimulant Type and Dose	ROS Regulatory Effects of Biostimulants Used	Reference
*Ficus carica* L. cv. Masui Dauphine	6 d	ALA (5 mg L^−1^) pretreatment	Leaf O_2_^•−^ production decreased by 62%. MDA contents were reduced. Enhanced SOD and POD activities.	[165]
*Triticum aestivum* L.	5 d	*Trichoderma asperellum* (strain MAP1) inoculums	Minimized the contents of MDA, H_2_O_2_, and EL. GSH content and activity of SOD and POD decreased.	[166]
*Triticum aestivum* L. Faisalabad-2008	7 d	Three Zn levels in seed: high (49 mg), medium (42 mg) and low (35 mg) kg^−1^ grain	Accumulation of MDA and antioxidant activity declined with the increase in intrinsic seed Zn levels.	[167]

## Data Availability

All information is presented in this article.
